# Masked Hypertension and Exaggerated Blood Pressure Response to Exercise: A Review and Meta-Analysis

**DOI:** 10.3390/diagnostics13061005

**Published:** 2023-03-07

**Authors:** Cesare Cuspidi, Elisa Gherbesi, Andrea Faggiano, Carla Sala, Stefano Carugo, Guido Grassi, Marijana Tadic

**Affiliations:** 1 Department of Medicine and Surgery, University of Milano-Bicocca, 20126 Milano, Italy; 2 Department of Cardio-Thoracic-Vascular Diseases, Foundation IRCCS Ca’ Granda Ospedale Maggiore Policlinico, 20122 Milan, Italy; 3 Department of Clinical Sciences and Community Health, University of Milano, 20122 Milan, Italy; 4 Department of Cardiology, University Hospital “Dr. Dragisa Misovic-Dedinje”, Heroja Milana Tepica 1, 11000 Belgrade, Serbia

**Keywords:** masked hypertension, hypertensive response to exercise, blood pressure, cardiovascular risk

## Abstract

Aim: Whether exaggerated blood pressure response (EBPR) to exercise represents a marker of masked hypertension (MH) in individuals with no prior history of hypertension is still unclear. We investigated this issue through a review and a meta-analysis of studies providing data on this association in normotensive individuals undergone both to dynamic or static exercise and to 24 h blood pressure monitoring (ABPM). Design: A systematic search was performed using Pub-Med, OVID, EMBASE, and Cochrane library databases from inception up to 31 December 2022. Studies were identified by using the following search terms: “masked hypertension”, “out-of-office hypertension”, “exercise blood pressure”, “exaggerated blood pressure exercise”, “exercise hypertension”. Results: Nine studies including a total of 387 participants with MH and 406 true normotensive controls were considered. Systolic BP (SBP) and diastolic BP (DBP) at rest were significantly higher in MH individuals than in sustained normotensives: 126.4 ± 1.4/78.5 ± 1.8 versus 124.0 ± 1.4/76.3 ± 1.3 mmHg (SMD: 0.21 ± 0.08, CI: 0.06–0.37, *p* = 0.007 for SBP; 0.24 ± 0.07, CI: 0.08–0.39, *p* = 0.002 for DBP). The same was true for BP values at peak exercise: 190.0 ± 9.5/96.8 ± 3.7 versus 173.3 ± 11.0/88.5 ± 1.8 mmHg (SMD 1.02 ± 0.32, CI: 0.39–1.65, *p* = 0.002 for SBP and 0.97 ± 0.25, CI: 0.47–1.96, *p* < 0.0001 for DBP). The likelihood of having an EBPR was significantly greater in MH than in their normotensive counterparts (OR: 3.33, CI: 1.83–6.03, *p* < 0.0001). Conclusions: Our meta-analysis suggests that EBPR reflects an increased risk of MH and that BP measurement during physical exercise aimed to assess cardiovascular health may unmask the presence of MH. This underscores the importance of BP measured in the medical setting at rest and in dynamic conditions in order to identify individuals at high cardiovascular risk due to unrecognized hypertension.

## 1. Introduction

Measurement of blood pressure (BP) in the medical environment by doctors or nurses still remains the most common procedure for diagnosing arterial hypertension [1]. In the last decades, however, growing evidence has been collected supporting the view that out-of-office BP measurements (i.e., home or ambulatory BP monitoring) are more reproducible than office ones, more closely related with sub-clinical hypertension-mediated organ damage (HMOD) as well as with the risk of non-fatal and fatal cardiovascular (CV) events [2,3,4,5]. Therefore, it is widely recognized that diagnosis of hypertension should be confirmed, when feasible, by out-of-office measurements [1,6]. Actual BP values may be either underestimated or overestimated by office BP measurements. In fact, the increasing combined performance of in- and out-office BP measurements allows to identify two opposite BP phenotypes such as white coat hypertension (i.e., the condition characterized by elevated BP in the office and by normal BP outside the medical environment) and masked hypertension (i.e., normal office BP but elevated out of office BP) [7,8]. These two BP patterns are quite common both in untreated individuals and in patients taking BP-lowering drugs, the prevalence rates being approximately 10–30% for white coat hypertension (WCH) and 10–15% for masked hypertension (MH) [9,10]. When WCH is not recognized in patients at low cardiovascular risk, antihypertensive treatment may be initiated or intensified in the absence of substantial benefits and with possible side effects of drugs. Differently, failure to recognize MH may have more serious consequences for CV health in the community, as this condition has been associated with a global CV risk similar to that of sustained hypertension [11,12,13]. Pierdomenico et al. [13] in their pioneering meta-analysis, performed in a pooled population of 7961 individuals who experienced a total of 696 CV events, reported that the group with MH exhibited a significantly higher risk of CV complications than the normotensive group (adjusted HR: 2.09, CI: 1.55–2.81, *p* < 0001), whereas this was not the case for the group with WCH (HR: 0.96, CI 0.65–1.42 *p* = 0.85).

As the systematic measurement of out-of-office BP in all members of the general population is unfeasible, the optimal screening strategy for MH remains uncertain, so far. Current hypertension guidelines state that the presence of out-of-office hypertension should be investigated by home or ambulatory BP monitoring (ABPM) in both untreated and treated individuals with office BP classified as high-normal (i.e., systolic 130–139 mmHg and/or diastolic 85–89 mmHg) [1,6].

In fact, high-normal office BP has been shown to entail an increased risk of MH especially when associated with comorbidities or conditions such as obesity, diabetes, obstructive sleep apnea, active smoking, and job stress [14,15,16]. Furthermore, some evidence indicates that an exaggerated BP response (EBPR) to exercise during electrocardiography (ECG)-monitored stress test for assessing cardiopulmonary fitness could be a marker of underlying MH [17]. Although the value of EPBR in predicting future hypertension and coronary artery disease, heart failure, and stroke has been consistently demonstrated in numerous clinical studies and meta-analyses, stress ECG testing with measurement of BP at incremental stages of exercise intensity is not recommended for hypertension work-up and for EBPR screening [18,19,20]. Starting from these premises which reflect the limits of current knowledge in this clinical field of great importance for public health, we sought to investigate through a meta-analysis whether and to what extent EBPR to exercise is associated with MH, in order to better understand whether the measurement of office BP during exercise may contribute to unmask MH, a condition which may lead to a marked increase in CV risk, if not timely identified.

## 2. Methods

The review was performed according to the key recommendations provided by Preferred Reporting Items of Systematic Reviews and Meta-Analyses (PRISMA) statement 2020. Medical literature was reviewed in order to identify all articles evaluating the relationship between MH, as defined by normal BP pre-exercise with elevated ambulatory BP, and EBPR. To this end, a systematic search was performed using Pub-Med, OVID, EMBASE, and Cochrane library databases from inception up to 31 December 2022. Studies were identified by using the following search terms: “masked hypertension”, “out-of- office hypertension”, “exercise blood pressure”, “exaggerated blood pressure exercise”, “hypertensive response to exercise”, “exercise hypertension”. Checks of the reference lists of selected papers and pertinent reviews complemented the electronic search. Data were examined and extracted by three independent investigators (C.C., A.F., and E.G.). In case of no agreement on a specific record, the full text of the study was analyzed by all reviewers in order to establish its eligibility according to the inclusion criteria mentioned below.

Main inclusion criteria were: (I) English articles published in peer-reviewed journals; (II) studies providing data on MH and EBPR; (III) minimum set of clinical/demographic data (i.e., sex, age, body mass index; office and/or ambulatory BP). Specific exclusion criteria were: (I) studies conducted in children and adolescents (age < 18 years); (II) case reports, reviews, and editorials. The Newcastle–Ottawa Scale (NOS) was used to measure the study quality (http://www.ohrica/programs/clinical_epidemiologyoxford.htm, accessed on 5 January 2023).

### Statistical Analysis

A pooled analysis of rest, exercise, and ambulatory BP values was performed using fixed or random effects meta-analysis by Comprehensive Meta-Analysis Version 2, Biostat, Englewood, NJ. Standard means difference (SMD) with 95% confidence interval (CI) was used to calculate the statistical difference between MH and normotensive individuals (i.e., demographic variables, rest and exercise, and ambulatory BP). Odds ratios (ORs) and 95% CI were calculated to assess differences in EBPR between the pooled groups. Demographic and clinical data provided by selected studies were expressed as absolute numbers, percentage, mean ± SD, mean ± SE or mean with CI. Heterogeneity was estimated using the I-squared test; random effect models were applied when heterogeneity across studies was high (I^2^ > 75). To assess the effect of individual studies on the pooled result, we conducted a sensitivity analysis by excluding each study one by one and recalculating the combined estimates on remaining studies. Publication bias was assessed by the Begg’s and Egger’s test. Statistical significance was set at *p* < 0.05.

## 3. Results

### 3.1. Search Results

**Figure 1:**
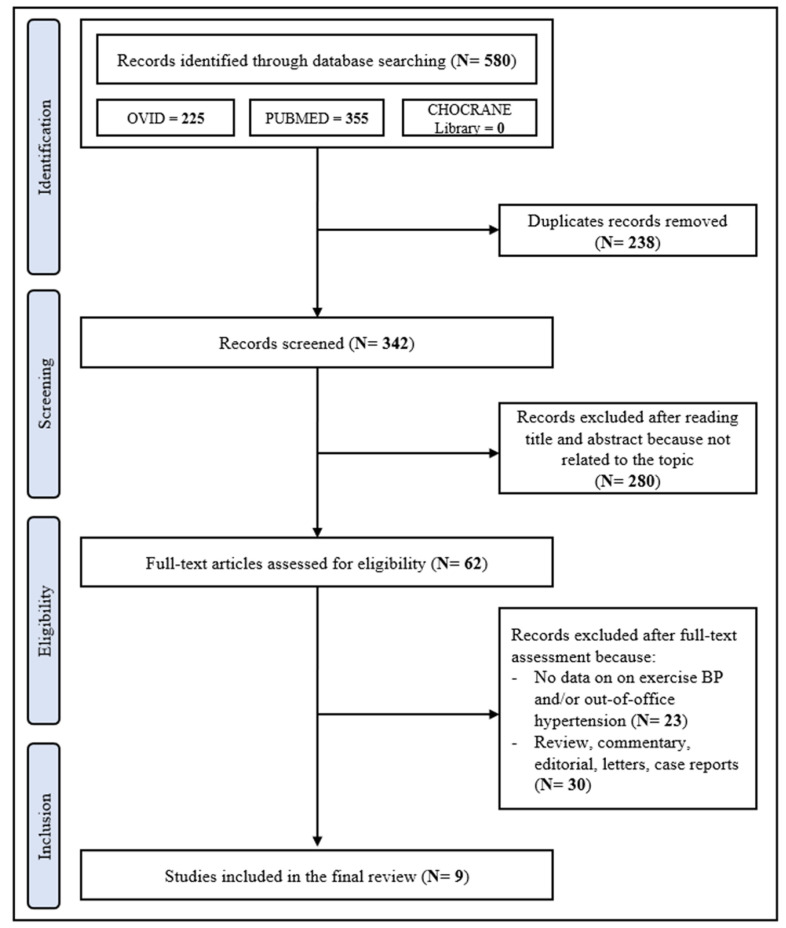
Schematic flow-chart for the selection of studies.

The PRISMA flowchart as presented in Figure 1 describes the full selection process. The first literature search identified 342 papers. After the initial screening of titles and abstracts, 280 studies were excluded as they were not related to the topic. Therefore, 62 studies were reviewed; of these, 23 did not report data on exercise BP and/or out-of-office hypertension, 30 were review, commentary, editorial articles, and case reports. A total of 9 studies addressing the relationship between EBPR and MH, as assessed by ABPM, were included in the analysis [21,22,23,24,25,26,27,28,29]. The Newcastle–Ottawa Score, used for assessing the quality of the studies, ranged from 7 to 9, the mean score being 7.6. Therefore, no study was excluded based on its limited quality.

### 3.2. Main Study Features

On the whole, 793 individuals were included in 9 studies (sample size ranging from 61 to 190 participants) performed in five countries (Australia = 1; Brazil = 1; Greece = 1; Israel = 1; Poland = 1; Turkey = 4).

Table 1 shows demographic and clinical characteristics of participants from selected studies such as sample size, mean age, prevalence of men, body mass index (BMI), setting, type of exercise, definition and prevalence rates of EBPR and MH. Mean age range was 41–54 years; 48% of participants were men. Average BMI ranged from 22.4 to 30.1 kg/m^2^. Seven studies included untreated healthy individuals. Two out of eight studies enrolled clinically normotensive patients with type 2 diabetes mellitus. An exercise test was performed in all studies but one with a treadmill. EBPR definition differed among studies, diagnostic thresholds for SBP ranging from 180 to 210 mmHg. In addition, gender-specific criteria and DBP values were considered by some but not all studies.

### 3.3. Demographic and Clinical Data in MH and Normotensive controls

The pooled group of 387 individuals meeting the diagnostic criteria for MH (48.8%) exhibited age, sex distribution, and BMI similar to normotensive controls’ values (age 50.3 ± 1.9 vs. 47.8 ± 1.9 years, SMD: 0.28 ± 0.24, CI:−0.19/0.75, *p* = 0.25; men prevalence 66 vs. 61%, *p* = 0.58; BMI 27.7 ± 1.2 vs. 27.7 ± 1.3 kg/m^2^, SMD: 0.10 ± 0.13, CI: −0.15/0.35, *p* = 0.44, respectively). Table 2 summarizes data regarding rest, exercise, and ambulatory BP (i.e., mean daytime and night-time) values of individuals with true normotension and MH as well as the prevalence of EBPR in the two groups.

**Figure 2:**
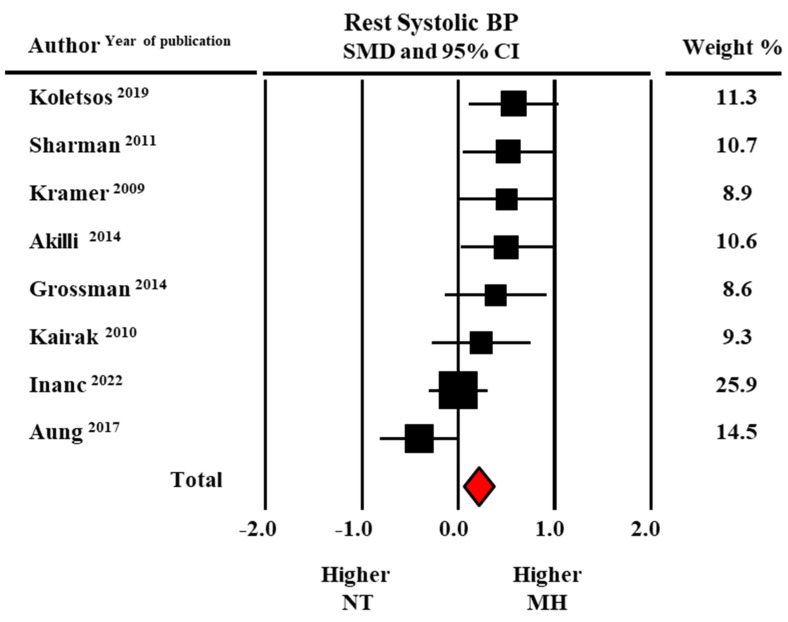
Forest plot of systolic blood pressure (BP) at rest in masked hypertensive (MH) and normotensive (NT) individuals. Standard mean difference (SMD) and 95% confidence interval (CI). Fixed model [21,22,23,24,25,26,27,29].

**Figure 3:**
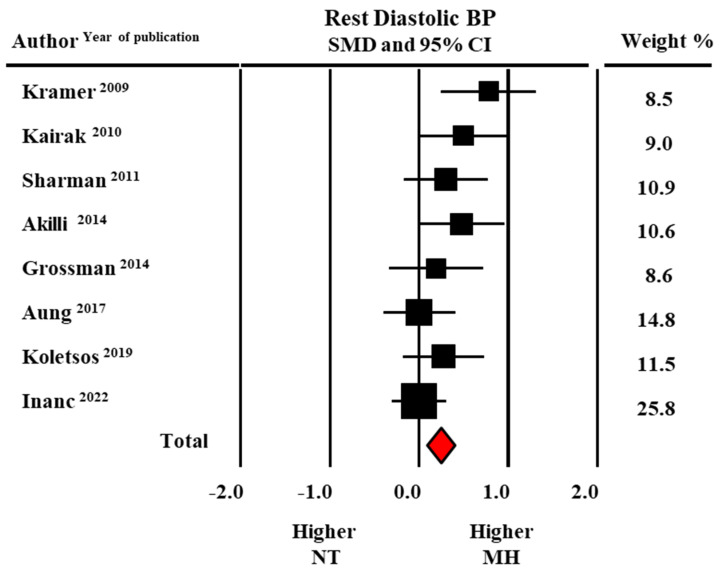
Forest plot of diastolic blood pressure (BP) at rest in masked hypertensive (MH) and normotensive (NT) individuals. Standard mean difference (SMD) and 95% confidence interval (CI). Fixed model [21,22,23,24,25,26,27,29].

Pooled average systolic BP (SBP) and diastolic BP (DBP) at rest were higher in individuals with MH than in sustained normotensives (126.4 ± 1.4/78.5 ± 1.8 versus 124.0 ± 1.4/76.3 ± 1.3 mmHg). As shown by the forest plot in Figure 2 and Figure 3, the meta-analysis of eight studies revealed a significant difference in SBP (SMD: 0.21 ± 0.08, CI: 0.06–0.37, *p* = 0.007) and DBP (SMD: 0.24 ± 0.07, CI: 0.08–0.39, *p* = 0.002) between groups.

**Figure 4:**
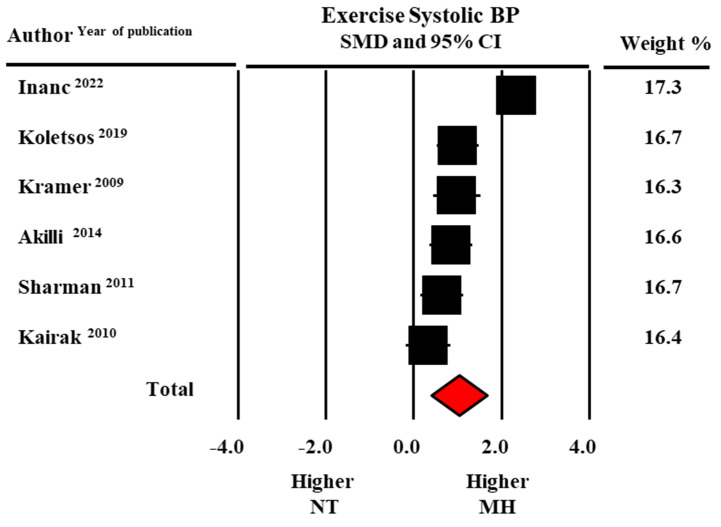
Forest plot of systolic blood pressure (BP) at peak exercise in masked hypertensive (MH) and normotensive (NT) individuals. Standard mean difference (SMD) and 95% confidence interval (CI). Fixed model [21,22,23,24,28,29].

**Figure 5:**
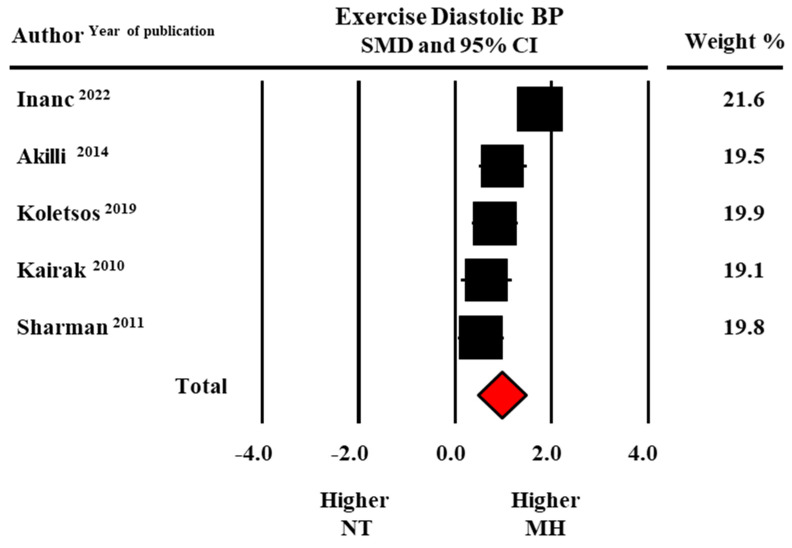
Forest plot of diastolic blood pressure (BP) at peak exercise in masked hypertensive (MH) and normotensive (NT) individuals. Standard mean difference (SMD) and 95% confidence interval (CI). Fixed model [22,23,24,28,29].

In addition, peak exercise SBP (190.0 ± 9.5 versus 173.3 ± 11.0 mmHg, data from six studies) and exercise DBP (96.8 ± 3.7 versus 88.5 ± 1.8 mmHg data from five studies) were higher in MH than in normotensive participants with statistically significant SMDs (1.02 ± 0.32, CI: 0.39–1.65, *p* = 0.002 for SBP and 0.97 ± 0.25, CI: 0.47–1.46, *p* < 0.0001 for DBP, respectively) (Figure 4 and Figure 5).

Findings provided by five studies showed that pooled daytime SBP and DBP values were greater in MH than in normotensive counterparts:142.9 ± 3.3/85.4 ± 1.3 vs. 120.6 ± 2.3/76.0 ± 1.7 mmHg (SMD: 2.69 ± 0.31, CI: 2.07–3.30, *p* < 0.0001 for SBP and 1.58 ± 0.11: 1.38–1.80, *p* < 0.0001, for DBP). This was also the case for night-time SBP and DBP: 129.1 ± 6.2/77.1 ± 3.8 vs. 108.1 ± 1.8/64.5 ± 0.9 mmHg, (SMD: 1.94 ± 0.47, CI: 0.99–2.97, *p* < 0.0001 for SBP and 1.59 ± 0.34: 0.92–2.26, *p* < 0.0001 for DBP).

**Figure 6:**
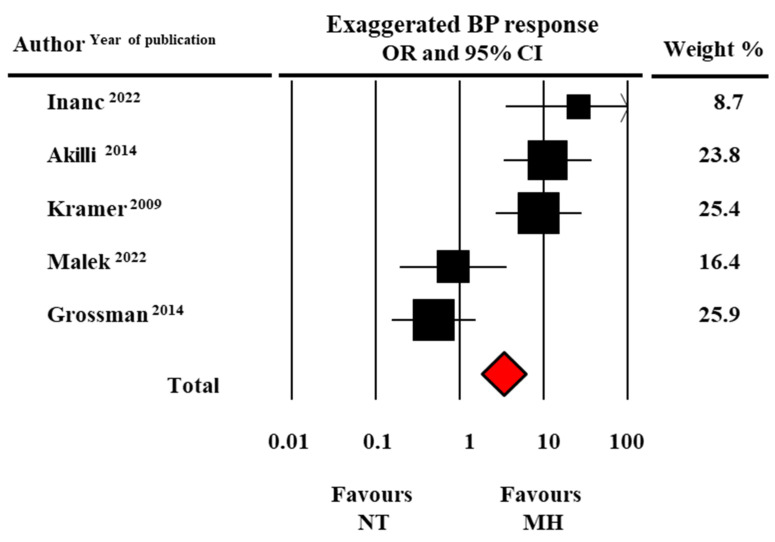
Forest plot of exaggerated systolic blood pressure (BP) to exercise in masked hypertensive (MH) and normotensive (NT) individuals. Odds ratio (OR) and 95% confidence interval (CI). Fixed model [21,24,25,28,29].

Finally, the likelihood of EBPR, assessed as an event rate, was found to be significantly higher in MH than in normotensive individuals: OR: 3.33, CI: 1.83–6.03, *p* < 0.0001 (data from five studies) (Figure 6).

### 3.4. Publication Bias

The presence of a single study effect was excluded at sensitivity analysis. An analysis by subgroups was performed by comparing studies including diabetic (two studies) vs. non-diabetic (n = 6 studies) patients. By this analysis, the odds ratio for EBPR was still significant in the diabetic subgroup (OR = 9.89, CI 1.2–23, *p* < 0.0001), but not in the non-diabetic subgroup (OR = 1.93, CI 0.2–15.7, *p* = 0.53). A publication bias was present for the following parameters: systolic BP at peak exercise and diastolic BP at rest and peak exercise.

## 4. Discussion

Our meta-analysis targeting the association between EBPR to exercise and MH, identified by 24 h ABPM, in untreated individuals without previously known hypertension provides the following main findings. Individuals fulfilling the MH diagnostic criteria had a three times greater risk of EBPR than true normotensives. Resting SBP and DBP measured at rest (before starting the exercise) were both significantly higher in the pooled MH group than in the normotensive group (approximately 2 mmHg for both SBP and DBP). Furthermore, differences between groups were even greater for SBP and DBP at peak exercise (about 17 mmHg higher for SBP and 8 mmHg for DBP in the MH group). Finally, higher ambulatory BP values in individuals with MH were not limited to daytime period but persisted during the night-time. This indicates that EBPR in MH does not exclusively reflect an increased BP reactivity to physical and psychic stimuli during daytime activities but also an altered nocturnal BP profile. Before addressing the details of our study, available evidence on this issue and related topics deserves be considered.

In healthy individuals, dynamic exercise during a stress electrocardiography test is associated to BP changes characterized by rapid, large increases in SBP and variable modifications in DBP (i.e., slight decrease or increase). Unfortunately, no consensus exists on EBPR metric, given the different diagnostic criteria recommended by major guidelines [30,31,32]. For instance, higher threshold values for SBP are recommended by European Society of Cardiology (ESC) guidelines (i.e., >220 mmHg in men and >200 mmHg in women) than those recommended by the American Heart Association (AHA) guidelines (i.e., >210 mmHg in men and >190 mmHg in women) [30,31]. Furthermore, differences between these two guidelines also concern threshold values for DBP which are, on the contrary, higher in AHA guidelines (>90 mmHg in both sexes) than in ESC ones (>85 mmHg in men and >80 mmHg in women).

These inconsistencies about the EBPR definition are widely witnessed by the heterogeneous criteria adopted in studies addressing this topic. A meta-analysis based on 12 studies including 53,264 participants revealed that SBP thresholds defining EBPR varied over a 90 mmHg wide range (i.e., from 180 to 275 mmHg) [20]. In line with these data, EBPR definition in the studies included in the present meta-analysis was based on different criteria either for SBP and DBP thresholds as well as gender specific BP thresholds.

Although the value of home BP monitoring (HBPM) as a reliable, cost-effective alternative to ABPM in the assessment of out-of-office BP has been universally recognized, MH screening should be preferentially based on ABPM, by using diagnostic criteria for both day- and night-time periods. When MH is defined according to HBPM (only based on daytime measurements) and ABPM daytime criteria, BP status may be misclassified, as nocturnal BP elevations may be missed. In this regard, it should be noted that six out of the eight studies included in the present meta-analysis did not consider average nocturnal BP separately from daytime or 24 h BP and this may have underestimated MH true prevalence, thus leaving isolated nocturnal hypertension to be unmasked.

Proposed pathophysiological mechanisms and clinical correlates of EBPR include familial predisposition to hypertension, endothelial dysfunction, impaired arterial baroreflex sensitivity, abnormal neurohormonal response to exercise, large artery stiffness, male gender, pre-hypertension, comorbidities such as obesity, diabetes, obstructive sleep apnea, and subclinical HMOD [33,34,35,36,37,38]. Over the last three decades, a large body of evidence has shown that all these factors, often associated, similarly also play a role in MH. Observational studies aimed at investigating clinical correlates of MH reported that male gender, older age, high normal office BP, active smoking, excessive alcohol drinking, obesity, metabolic syndrome, job stress, and sleep apnea syndrome are major factors responsible of out-of-office hypertension in clinically normotensive individuals. For instance, participants with MH in the Pensioni Arteriose Monitorate e Loro Associazioni (PAMELA) study [37] showed a greater male prevalence, age, BMI, office and 24 h heart rate, and office and 24 h mean SBP/DBP compared to participants with true normotension [39]. In contrast, our meta-analysis revealed that participants with MH had similar clinical features as normotensives. In fact, no statistically significant difference was found in age, gender distribution, and BMI between the pooled groups. This allows us to hypothesize that EBPR increased risk in MH participants may occur independently of these demographic and clinical variables.

It should be underlined, however, that the pooled group with MH had significantly higher resting office BP values (i.e., measured before the onset of exercise) than the normotensive group. The significance of office BP at rest as a predictor of MH deserves a more in-depth comment. In the Masked Hypertension Study [16], a population-based study including 769 participants with normal office BP levels, MH prevalence (daytime BP >135/85 mmHg) was 15%. Of note, this condition was rarely detected (4%) when office BP was optimal (<120/80 mmHg); conversely, 84% of MH participants had pre-hypertension (office BP 120–139/80–89 mmHg). The overlap between MH and pre-hypertension has been also documented by studies where this BP pattern was diagnosed by home BP monitoring. Findings from investigations carried out in treated hypertensive cohorts and in community-based samples showed that office BP in the pre-hypertensive range is associated with MH [40,41,42]. More recently, a close relation between MH, assessed by home BP monitoring, and pre-hypertension has been reported in a group of 703 untreated normotensive individuals [43]. MH was found in 20.6% of the whole sample and in over a quarter of participants with BP values between 135/85 and 139/89 mmHg. The prevalence of MH depends on the methods used to assess out-of-office BP (home vs. ABPM), diagnostic thresholds (average 24 h vs. daytime and/or night-time BP) as well as on the clinical characteristics of the setting examined (i.e., general population, individuals with suspected hypertension or comorbidities). In our series, MH accounted for almost half of the participants, a much higher percentage than that reported by the majority of published studies [44]. One possible explanation is that five out of nine studies considered in the present meta-analysis included individuals not representative of the general population, as three of them enrolled only subjects with EBPR and two of them diabetic patients.

A further important result of our meta-analysis was that BP recorded at peak exercise was markedly higher in individuals with MH than in true normotensives, the difference between groups being 17 mmHg for SBP and 8 mmHg for DBP, resulting in a three times higher risk of EBPR in the MH group. Increased adrenergic activity, in addition to abnormal neurohormonal response to exercise, subclinical organ damage such as increased arterial stiffness, and cardiac remodeling are thought to be key factors associated with EBPR.

Although a discussion about the possible mechanisms of EBPR in patients with MH is beyond the scope of this meta-analysis, some aspects of this topic deserve to be briefly highlighted. Four of the studies included in our meta-analysis provided data on cardiac HMOD [23,26,27,28,29]. Sharman et al. showed that patients with MH had higher values of LV mass index (42 ± 9 vs. 36 ± 9 g/m^2^.^7^) and relative wall thickness (0.42 ± 0.1 vs. 0.37 ± 0.1) than their normotensive counterparts [23]. Similarly, a worse cardiovascular risk profile, as reflected by increased LV mass index, left atrial volume, ticker interventricular septum, and higher pulse wave velocity was found in MH individuals by Aung et al. [26] and Koletsos et al. [27]. Finally, the study by Malek et al. [28], including 71 healthy, male master athletes revealed that LVH prevalence was markedly higher in the subgroup with MH (42%) than in the normotensive one with normal EBPR (16%). These findings are in keeping with a large meta-analysis based on 49 studies including 23,707 normotensive and hypertensive individuals which documented that exercise SBP had a direct and linear relationship with LV mass index, relative wall thickness, LV posterior wall thickness, interventricular septum thickness, and left atrial diameter [45].

The value of resting heart rate as an indicator of sympathetic overactive state and as a predictor of adverse CV prognosis has been widely recognized for many decades [46]. In this respect, none of the studies included in the present meta-analysis showed significantly higher resting heart rates in individuals with MH compared to normotensives (data not shown). It should be remarked, however, that indirect approaches for assessing sympathetic function (i.e., resting heart rate by ECG) have a lower diagnostic sensitivity than more refined, direct techniques such catecholamine spillover and microneurographic nerve recordings [47]. A pioneering study by Grassi et al. [48] documented that patients with sustained hypertension, MH, and WCH displayed a resting sympathetic nerve activity measured by microneurography significantly greater than normotensive subjects.

A further point should be made. MH may present different clinical patterns such as persistent out-of-office hypertension, isolated daytime, or isolated nocturnal hypertension [49]. Information on this issue, based on four of the studies included in the meta-analysis, revealed that MH patients (identified according to daytime BP criteria) had significantly higher nocturnal BP values than controls. This finding, therefore, underlines that EBPR can also be a marker of nocturnal hypertension of poor prognostic significance [50]. Finally, it should be noted that the subgroup meta-analysis revealed that the association between EBPR and MH maintained its statistical significance in patients with diabetes, but not in non-diabetic individuals. Although these data must be taken with caution, it is possible to hypothesize that the hypertensive response to exercise in diabetics may be a robust indicator of MH.

### Limitations

Some limitations of our meta-analysis deserve to be reported. Our search was restricted to studies published in English due to the problems in identifying and interpreting papers in other languages. This linguistic approach may have affected our results. The present findings are based on few studies conducted in different settings including a few hundred cases and controls. As a consequence of this, the power of various tests to evaluate publication bias is too low to provide reliable results on this issue. Diagnostic criteria used to define EBPR and MH were heterogeneous and this may have influenced the results. The observational nature of the studies included in the meta-analysis does not allow to draw any conclusion on the causal relationship between MH and EBPR. It should be emphasized that both MH and EBPR are not seen as reproducible clinical traits over time [25,51,52]. The strengths refer to the fact that out-of-office BP was assessed by ABPM in all studies; moreover, only untreated individuals, therefore, truly “masked” hypertensives were included.

## 5. Conclusions

Our meta-analysis suggests that EBPR portends the likely presence of MH, a BP phenotype which, if not identified, carries a CV risk similar to that of sustained hypertension. This implies that measuring BP in the medical setting during stress testing offers a unique opportunity to identify individuals at high risk of MH. In a clinical perspective, the occurrence of EBPR during an ECG stress test should direct the clinician to plan investigations to unmask the presence of MH and subclinical HMOD in order to reduce the risk of CV complications in this non-marginal fraction of the general population.

## Figures and Tables

**Table 1:** Summary of nine studies that addressed the relationship between exaggerate blood pressure response to exercise and masked hypertension. Data are presented as absolute numbers, percentage, mean ± SD. BMI = body mass index; EBPR = exaggerated blood pressure response to exercise; MH = masked hypertension.

Author [Reference] Year Publication	Type of Study	Sample Size (n)	Men (%)	Age (years)	BMI (kg/m^2^)	Setting	Exercise Testing	EBPR Definition	EBPR (%)	MH (%)
Kramer [21] 2009	Observational cross-sectional case-control study	61	49	53 ± 9	28.2 ± 4.2	DM	Treadmill	SBP > 180 mmHg	41	39
Kairak [22] 2010	Observational cross-sectional study	61	79	47 ± 10	28.5 ± 4.5	Healthy subjects with EBPR	Treadmill	SBP ≥ 210 (M), ≥190 mmHg (F)	100	41
Sharman [23] 2011	Observational cross-sectional study	72	60	54 ± 9	28.6 ± 3.9	Healthy subjects with EBPR	Treadmill	BP ≥ 210/105 (M), ≥190/105 mmHg (F)	100	58
Akilli [24] 2014	Observational cross-sectional study	85	62	51 ± 8	30.1 ± 5.1	DM	Treadmill	BP > 200/100 mmHg	13	28
Grossman [25] 2014	Retrospective observational cross-sectional study	69	87	54 ± 9	27.2 ± 3.1	Healthy subjects with high-normal BP	Treadmill	SBP ≥ 200 mmHg	62	72
Aung [26] 2017	Observational cross-sectional study	98	62	41 ± 6	27.3 ± 1.9	Healthy subjects with EBPR	Treadmill	SBP > 200 (M), >190 mmHg (F)	100	40
Koletsos [27] 2019	Observational cross-sectional study	86	56	46 ± 10	27.0 ± 4.2	Hypertensive and normotensive subjects	Handgrip	n. a.	n.a.	31
Malek [28] 2022	Observational cross-sectional study	71	100	41 ± 6	24.5 ± 3.0	Athletes	Treadmill	SBP > 210 mmHg	30	37
Inanc [29] 2022	Observational cross-sectional study	190	45	59 ± 10	22.3 ± 1.2	Masked hypertensive and normotensive subject	Treadmill	SBP ≥ 210 (M), ≥190 mmHg (F)	22	68

**Table 2:** Rest, exercise, and ambulatory blood pressure values of individuals with true normotension and masked hypertension in nine studies targeting exaggerated blood pressure response to exercise. Data are presented as absolute numbers, percentage, mean ± SD, BMI = body mass index; BP = blood pressure; EBPR = exaggerated blood pressure response to exercise; MH = masked hypertension; NTN = normotension.

	OFFICE BP at Rest (mmHg)		DAY-TIME BP (mmHg)		NIGHT-TIME BP (mmHg)		EXERCISE BP (mmHg)		EBPR (%)	
Author [Reference] Publication Year	NTN	MH	NTN	MH	NTN	MH	NTN	MH	NTN	MH
Kramer [21] 2009	122 ± 9/74 ± 6	126 ± 6/79 ± 7	n.a.	n.a.	n.a.	n.a.	167 ± 16	185 ± 22	21	71
Kairak [22] 2010	126 ± 13/84 ± 10	129 ± 12/ ± 89 ± 10	123 ± 7/77 ± 5	142 ± 7/87 ± 6	111 ± 8/66 ± 7	133 ± 10/81 ± 8	209 ± 19/91 ± 9	214 ± 9/98 ± 13	100	100
Sharman [23] 2011	121 ± 9/72 ± 6	126 ± 10/74 ± 7	127 ± 6/78 ± 5	142 ± 8/86 ± 6	112 ± 5/67 ± 5	119 ± 6/71 ± 5	212 ± 14/90 ± 10	222 ± 17/96 ± 12	100	100
Akilli [24] 2014	121 ± 8/77 ± 6	125 ± 8/80 ± 7	122 ± 4/78 ± 5	140 ± 5/84 ± 4	115 ± 10/65 ± 6	131 ± 13/74 ± 6	155/83	168/87	8	25
Grossman [25] 2014	129 ± 7/81 ± 6	132 ± 8/82 ± 5	n. a.	n. a.	n.a.	n.a.	n. a.	n. a.	73	58
Aung [26] 2017	130 ± 9/73 ± 5	126 ± 11/73 ± 5	n. a.	n.a.	n.a.	n.a.	n. a.	n. a.	100	100
Koletsos [27] 2019	123 ± 9/79 ± 7	128 ± 8/81 ± 8	121 ± 8/76 ± 6	137 ± 7/86 ± 6	106 ± 9/63 ± 6	116 ± 10/70 ± 7	160 ± 16/91 ± 9	175 ± 13/98 ± 7	n. a.	n. a.
Malek [28] 2022	n. a.	n. a.	n. a.	132 ± 7/80 ± 5	n.a.	n.a.	n. a.	n. a.	13	51
Inanc [29] 2022	120 ± 15/72 ± 11	120 ± 15/72 ± 11	111 ± 9/73 ± 4	164 ± 22/106 ± 15	97 ± 9/62 ± 4	147 ± 16/89 ± 13	140 ± 8/98 ± 11	177 ± 18/103 ± 11	2	31

## Data Availability

Data are available upon request.
